# Metastasis of Colorectal Adenocarcinoma to the Thyroid: A Case Report and Review of the Literature

**DOI:** 10.1155/2012/179407

**Published:** 2012-11-28

**Authors:** C. Goatman, P. J. Goldsmith, V. Antonopoulos, B. Ali

**Affiliations:** Department of Colorectal Surgery, North Manchester General Hospital, Delaunays Road, Crumpsall M8 5RB, UK

## Abstract

*Purpose*. We present a rare case of colorectal metastasis to the thyroid five years following primary colonic resection. This case highlights the need to be cognisant of unusual sites of metastasis from colorectal neoplasms. *Case Report*. An 82-year-old male patient had a panproctocolectomy for synchronous colorectal tumours. Five years later he was found to have lung and thyroid metastases found incidentally on imaging for an acute presentation with small bowel obstruction. *Conclusion*. Metastases to the thyroid should be considered in the differential diagnosis of the thyroid lesion with any history of malignancy, particularly with increasing patient age and when renal cell carcinoma or lung, colon, or breast primaries are involved.

## 1. Introduction

Metastases to the thyroid are rare. Clinical incidence has been reported as between 1.4–3% of all patients operated on for suspected thyroid malignancy [1–3]. However rates of up to 24% in autopsy series of patients who die as a result of cancer have been reported [4], suggesting they may be more common than is clinically apparent. One UK centre reports thyroid metastases making up 1.5% of patients undergoing surgery for thyroid malignancy over a 17-year period [5].

We report a case of an 82-year-old male with a history of colorectal cancer, with a thyroid lesion found incidentally on CT scan. Incidence and prognosis of patients with thyroid metastases are discussed and literature reviewed. This case highlights that thyroid metastases should be considered in patients presenting with a thyroid nodule, with any history of neoplasm, especially of the kidney, lung, breast, and gastrointestinal tract, and particularly with increased patient age [2, 3].

## 2. Case History

An 82-year-old male patient was admitted acutely with a small bowel obstruction. Five years previously he had undergone a panproctocolectomy for synchronous colorectal cancer of the caecum and rectum. The patient declined postoperative chemotherapy. 

**Figure 1:**
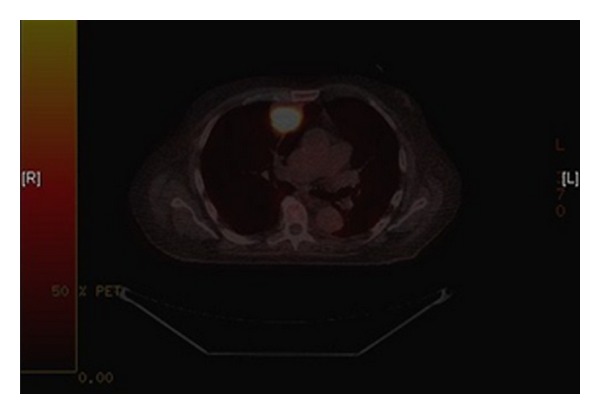
PET image showing avid lesion in the right lung.

The small bowel obstruction was treated conservatively and resolved uneventfully. A computerised tomography (CT) scan performed as part of the acute investigation incidentally revealed a right upper lobe lung opacity which was avid on subsequent positron emission tomography (PET) scanning (Figure 1). On CT guided biopsy this was shown to be an adenocarcinoma contacting the pleura, histopathologically consistent with metastatic deposit from a colonic primary, with further tiny right lung nodules of minimal PET avidity also seen.

**Figure 2:**
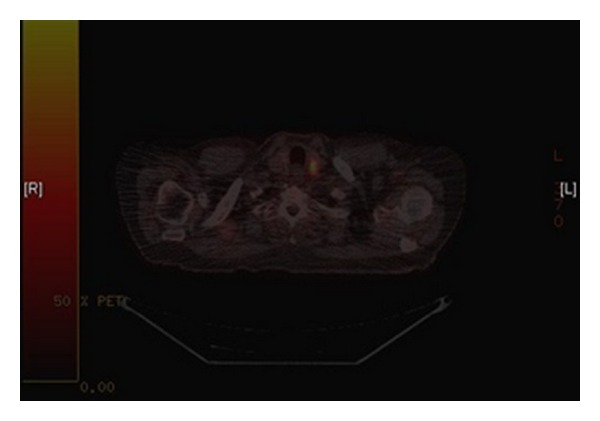
PET image showing thyroid nodule.

PET scan also revealed a left thyroid nodule (Figure 2). The patient was euthyroid throughout. Following multidisciplinary team (MDT) discussion an ultrasound (USS) and fine needle aspiration biopsy (FNAB) were performed on the thyroid nodule. Thyroid FNA was suspicious for papillary cell carcinoma with THY5 on immunohistochemistry.

The patient underwent a right lung upper lobectomy initially, from which he made a good recovery. Three months later he went on to have a total thyroidectomy and left neck dissection. Intraoperatively the tumour was found to involve the recurrent laryngeal nerve, which had to be sacrificed. Histological examination of the excised thyroid gland found a metastatic adenocarcinoma with papillary features, consistent with a metastatic colonic adenocarcinoma. The lesion occupied almost the entire left lobe of the thyroid, with extension into the extra thyroid soft tissue including skeletal muscle. There was no involvement of any of the sampled nodes. The patient made a good recovery postoperatively.

## 3. Discussion

Metastatic deposits occur to the thyroid gland due to vascular or lymphatic spread. It has been postulated that the rich vasculature of the thyroid makes it particularly liable [6], but also that the fast flow of blood through the gland may reduce the likelihood of metastatic deposits [3]. Metastases to the thyroid from nonthyroid malignancies (TM) remain a rare occurrence in clinical practice, comprising only 1.4–3% of all thyroid neoplasms [4].

Studies from the US and UK [5, 7] place kidney, followed by colorectal, lung, and breast as the most common primary sources for thyroid metastases, while an Asian series indicate malignancies of the gastrointestinal tract, especially of the upper GI tract, are also responsible [7]. Other reported primary tumours include neuroendocrine tumours, sarcoma, and more rarely melanoma [2, 3].

The timing of diagnosis of metastases to the thyroid is variable from time of initial diagnosis to years after treatment. The majority of patients are euthyroid at presentation, with thyrotoxicosis rarely seen [7]. When it does occur it is thought to be due to hormones leaking from the gland following neoplastic damage [3]. Metastases to the thyroid can present as a single nodule or as multiple foci within the gland [4]. 72% of patients with metastases to the thyroid in one study presented with a clinically detectable thyroid nodule [7], with other studies reporting signs of extrinsic compression at presentation [2, 3, 5]. In contrast to the much greater preponderance of primary thyroid tumours in women than men, the picture regarding metastases of nonthyroid malignancies to the thyroid gland is less clear. Some studies have reported a slightly higher incidence in women than men [5], but other series report no sex bias [7]. A recent review of the last 10 years suggests a female to male ratio of 1.4 : 1 [3].

Investigation of TM usually proceeds as with the assessment of any thyroid nodule-history, clinical examination, followed by USS and FNAB, with CT and PET imaging also playing a role [8]. Metastases to the thyroid typically appear ultrasonographically as hypoechoic masses with poorly defined margins and increased vascularisation. Calcification is rarely seen, in contrast to many primary thyroid tumours [7]. Immunohistochemistry is key in the diagnosis of metastases to the thyroid and the identification of the primary site. A nonthyroidal primary site may be indicated by a failure to react to thyroglobulin, TTF-1, and calcitonin [7], and indeed metastatic thyroid tumours are almost universal in their failure to react to thyroglobulin [6]. It is important to note however that the majority of anaplastic thyroid carcinomas also do not have a positive reaction to thyroglobulin [7].

It has been suggested in more recent studies over the last 10 years that overall survival time is not significantly different in patients with or without thyroid metastases [7], which differs from series published prior to 2000 [9]. The impact of metastases to the thyroid on disease course may be the same as nonthyroid metastases, with prognosis dependent on the primary tumour and disease [2, 3, 7, 8]. Interestingly, there has been no significant effect on overall survival shown following thyroidectomy in patients with TM [7].

## 4. Conclusion

The case presented and literature reviewed suggests that although thyroid metastases are a rare occurrence there should be a high index of suspicion in patients with a history of malignancy presenting with a thyroid lesion [4], regardless of the time elapsed between diagnosis and treatment of the primary tumour and regardless of whether this treatment was considered to be curative. Thyroid metastases should be considered in the differential diagnosis with any history of malignancy, particularly with increasing patient age and when renal cell carcinoma, lung, colon, or breast primaries are involved.
